# Copper Chaperone for Cu/Zn Superoxide Dismutase is a sensitive biomarker of mild copper deficiency induced by moderately high intakes of zinc

**DOI:** 10.1186/1475-2891-4-35

**Published:** 2005-11-24

**Authors:** Monica Iskandar, Eleonora Swist, Keith D Trick, Bingtuan Wang, Mary R L'Abbé, Jesse Bertinato

**Affiliations:** 1 Nutrition Research Division, Food Directorate, Health Products and Food Branch, Health Canada, 2203C Banting Research Centre, Ottawa, ON, K1A 0L2, Canada

## Abstract

**Background:**

Small increases in zinc (Zn) consumption above recommended amounts have been shown to reduce copper (Cu) status in experimental animals and humans. Recently, we have reported that copper chaperone for Cu/Zn superoxide dismutase (CCS) protein level is increased in tissues of overtly Cu-deficient rats and proposed CCS as a novel biomarker of Cu status.

**Methods:**

Weanling male Wistar rats were fed one of four diets normal in Cu and containing normal (30 mg Zn/kg diet) or moderately high (60, 120 or 240 mg Zn/kg diet) amounts of Zn for 5 weeks. To begin to examine the clinical relevance of CCS, we compared the sensitivity of CCS to mild Cu deficiency, induced by moderately high intakes of Zn, with conventional indices of Cu status.

**Results:**

Liver and erythrocyte CCS expression was significantly (*P *< 0.05) increased in rats fed the Zn-60 and/or Zn-120 diet compared to rats fed normal levels of Zn (Zn-30). Erythrocyte CCS expression was the most sensitive measure of reduced Cu status and was able to detect a decrease in Cu nutriture in rats fed only twice the recommended amount of Zn. Liver, erythrocyte and white blood cell CCS expression showed a significant (*P *< 0.05) inverse correlation with plasma and liver Cu concentrations and caeruloplasmin activity. Unexpectedly, rats fed the highest level of Zn (Zn-240) showed overall better Cu status than rats fed a lower level of elevated Zn (Zn-120). Improved Cu status in these rats correlated with increased duodenal mRNA expression of several Zn-trafficking proteins (i.e. MT-1, ZnT-1, ZnT-2 and ZnT-4).

**Conclusion:**

Collectively, these data show that CCS is a sensitive measure of Zn-induced mild Cu deficiency and demonstrate a dose-dependent biphasic response for reduced Cu status by moderately high intakes of Zn.

## Background

Zinc (Zn) and copper (Cu) play vital roles as structural and catalytic components of metalloenzymes and are essential nutrients required for growth and development [1-3]. It is well recognized that consuming large quantities of Zn for extended periods of time causes severe Cu deficiency and can lead to the development of anaemia and other abnormalities [4-8]. Of potentially greater significance, however, are studies showing that even small increases in Zn consumption above recommended amounts depress Cu status in experimental animals [9,10] and humans [11,12]. This strong antagonism of excess Zn to Cu status was the basis for setting the Tolerable Upper Intake Levels (ULs) for Zn [1]. At present, the mechanism by which Zn impedes Cu absorption is not known.

In humans, overt Cu deficiency is uncommon and usually only seen in specific situations [13]. However, mild Cu deficiency from consuming diets inadequate in Cu [14,15] or high in Zn [16] may be of concern. Notably, studies have shown that a large proportion of young children have Zn intakes exceeding the ULs and much of the Zn consumed by these children is from Zn-fortified foods [16-18]. Although overt Zn toxicity has not been reported in these children, excessive Zn intake may cause small but potentially harmful reductions in Cu balance, stressing the need for sensitive biomarkers able to detect mild Cu deficiency.

Insertion of Cu into Cu/Zn superoxide dismutase (SOD1) requires the copper chaperone for SOD1 (CCS). We have previously shown that CCS protein is up-regulated in liver and erythrocytes of Cu-deficient rats [19] and Cu regulates the degradation of CCS by the 26 S proteosome [20]. Another group has since reported a similar increase in CCS in tissues of Cu-deficient rats and mice [21,22]. CCS is a promising biomarker of reduced Cu status, as CCS protein level is more responsive to Cu deficiency than reduction in SOD1 activity [19], the endpoint used to set the ULs for Zn [1]. Further, CCS is particularly appealing given that commonly used measures of Cu status are affected by various common conditions unrelated to Cu nutriture [14,15,23-25] and thought to be insensitive to marginal deficiency [14,15,26]. The latter is underscored by experiments in rats showing that diets marginally low in Cu induce abnormalities in heart morphology and function, but minimal changes in conventional indices of Cu status [27,28].

Several reports have established increased CCS protein in tissues of overtly Cu-deficient rats and mice [19-22], however, it is not known whether CCS is responsive to small reductions in Cu status. The objectives of this study were two-fold; (1) to examine the effects of graded levels of moderately high dietary Zn on Cu status and (2) to evaluate the ability of CCS to detect small reductions in Cu nutriture, induced by excess Zn, as a first step in determining the usefulness of CCS as a biomarker that can be used in a clinical setting.

## Methods

### Animals and Test Diets

Weanling (21-day-old) male Wistar rats (*n *= 12/diet group) (Charles River Canada, St. Constant, Canada) had free access to one of 5 test diets [29] and demineralised drinking water. Food consumption and body weight were measured weekly. After 5 weeks of consuming the diets, rats were killed by exsanguination while anesthetised with 3% isoflurane. Blood was withdrawn from the abdominal aorta. The intestine was extracted and intestinal contents removed by washing with isotonic saline. Extracted intestine, liver and kidneys were frozen until analysis. The Health Products and Food Branch Animal Care Committee of Health Canada approved the experimental protocol. Rats were treated in accordance with the guidelines of the Canadian Council on Animal Care.

Test diets were prepared by adding appropriate amounts of Cu (cupric carbonate) and Zn (zinc carbonate) from cornstarch premixes. Other than differences in Cu and Zn content, composition of the diets were similar to those described previously [19]. Zn and Cu content in samples of each test diet were determined by flame atomic absorption spectrophotometry (AAS) (Perkin-Elmer 5100 PC; Perkin Elmer Cetus Instruments, Norwalk, CT) as described [30].

### Blood Fractionation

Blood samples were collected in EDTA tubes and separated into its components by centrifugation. Plasma was frozen in aliquots. White blood cells (WBCs) were carefully removed (trying to avoid contamination with erythrocytes), washed with isotonic saline and centrifuged. The procedure was repeated 3–4 times until a WBC preparation with no visible contamination with erythrocytes was obtained. Erythrocytes were washed 3 times with saline prior to freezing.

### Haematological Measurements

Blood samples were collected in vacutainer K_3_EDTA tubes and shipped to VITA-TECH (Markham, Canada) for analysis of haematological parameters (See additional file 1: Table 1).

**Table 1:** Total food consumption, body weight and Zn content in tissues of rats fed diets differing in Zn and Cu^1,2^

Diet Group	Test Diets (mg/kg diet)		Total Food Consumption (g)	Body Weight week 5 (g)	Liver Zn (μg/g dry weight)	Kidney Zn (μg/g dry weight)	Mucosal Zn (μg/g dry weight)
						
	Zn	Cu					
Zn-30	41.27 ± 0.52^a^	5.70 ± 0.04^a^	881.0 ± 20.8^a^	345.1 ± 6.2^a^	91.99 ± 1.99^a^	104.82 ± 1.80^a^	97.12 ± 2.14^a^
Zn-60	65.09 ± 3.42^b^	5.57 ± 0.14^a^	876.9 ± 28.6^a^	346.3 ± 7.3^a^	94.08 ± 1.20^a^	102.42 ± 1.36^a^	98.94 ± 1.31^a^
Zn-120	129.18 ± 0.71^c^	5.77 ± 0.08^a^	852.8 ± 26.6^a^	343.4 ± 6.4^a^	104.40 ± 1.91^b^	105.95 ± 1.05^a^	115.28 ± 2.80^b^
Zn-240	242.22 ± 0.80^d^	5.78 ± 0.03^a^	908.6 ± 28.7^a^	364.9 ± 9.0^b^	109.33 ± 2.25^b^	112.97 ± 2.19^b^	146.04 ± 6.77^c^
Cu-D	48.48 ± 2.54^e^	1.07 ± 0.02^b^	891.2 ± 28.3^a^	339.0 ± 8.5^a^	94.69 ± 1.53^a^	102.07 ± 0.77^a^	94.93 ± 1.38^a^

^1 ^Values in a column without a common letter differ, *P *< 0.05.

^2 ^Values are means ± SEM, n = 12/diet group.

### Western Blotting

Liver protein extracts were prepared by homogenizing in ice-cold 0.5% (v/v) Triton-X-100 buffer containing a protease inhibitor cocktail (Roche, Laval, Canada). WBCs and erythrocytes were lysed in Triton-X-100 buffer or GSH reagent (5 mmol/L KH_2_PO_4_/K_2_HPO_4_, 2 mmol/L glutathione, pH 7.0), respectively. Extracts (40 μg total protein or haemoglobin) were separated over 8–16% Tris-Glycine gradient gels (Invitrogen, Burlington, Canada) under denaturing and reducing conditions. Gels for each tissue were simultaneously electroblotted onto a single nitrocellulose membrane. The membrane was blocked for 1 h at room temperature (RT) in TBS-Tween [20 mmol/L Tris, 500 mmol/L NaCl, 0.1% Tween 20 (v/v), pH 7.5] supplemented with 5% (wt/v) nonfat dry milk (BioRad, Hercules, CA). Membranes were probed with a CCS antibody (FL-274; Santa Cruz Biotechnology, Santa Cruz, CA) at a final concentration of 0.6 mg/L (for liver and erythrocytes) or 0.2 mg/L (for WBCs) overnight at 4°C. After washing with TBS-Tween, membranes were incubated with an anti-rabbit (0.16 mg/L) HRP-conjugated secondary antibody (BioRad) in blocking solution for 2 h at RT. Antibody-bound proteins were detected by enhanced chemiluminescence and exposure to film. Membranes were stripped with stripping buffer (100 mmol/L 2-mercaptoethanol, 2% (wt/v) SDS, 62.5 mmol/L Tris-HCl, pH 6.8) and re-probed with an antibody against Actin [(I-19)-R, Santa Cruz Biotechnology] or GAPDH (MCA-1D4, Encor Biotech, Alachua, FL) at 0.5 mg/L concentration or at a 1:1000 dilution, respectively. Film was scanned and band intensity determined using Scion Image software (Scion Corporation, Frederick, MD). Band intensities were determined at exposures within the linear response range of the film.

### Mineral Analyses in Tissues

Cu and Zn content in liver and kidney samples were determined by flame AAS as described [30]. Plasma Cu concentration was measured by graphite furnace AAS using a SIMAA 6000 (Perkin-Elmer Cetus Instruments) with Zeeman background correction. To determine Zn and Cu levels in the intestinal mucosa, mucosal cells were gently scraped with a glass cover slide from a 10 cm intestinal segment starting 10 cm caudal to the pyloric sphincter. Mucosal scrapings were dried, dissolved in concentrated HNO_3 _and microwave digested using a CEM (Mars5) microwave (CEM Corporation, Matthews, NC). Zn and Cu in digested samples were determined by flame AAS and graphite furnace AAS, respectively.

### Quantitative PCR

A segment (1 cm in length; starting 8 cm caudal to the pyloric sphincter) of frozen intestine was excised and total RNA isolated using QIAGEN's RNeasy mini kit (QIAGEN, Mississauga, Canada). All RNA samples were treated with RNase-free DNase (QIAGEN). cDNA from each sample was generated using an oligo (dT) primer (Ambion, Austin, TX). Sequences for genes (*MT-1*, *ZnT-1*, *ZnT-2 *and *ZnT-4*) were obtained from GenBank (See additional file 2: Table 2 QPCR primers). To obtain the rat *Zip4 *sequence, primers specific for the mouse *Zip4 *gene (Forward: 5'-ACT GGA CGG CCT GTT AAA TAC GCT-3'; Reverse: 5'-TAC TCC GAC TGC TAG AGC CAC GTA-3') were used to amplify the rat *Zip4 *cDNA from total RNA. The sequence of the PCR product was aligned against the rat genome and the mouse *Zip4 *gene to confirm amplification of the rat *Zip4 *cDNA. All primers used for QPCR (See additional file 2: Table 2 QPCR primers) were designed using PrimerQuest (Integrated DNA Technologies, Skokie, IL). QPCR was performed using an Mx4000 Multiplex Quantitative PCR System (Stratagene, La Jolla, CA). Reactions were performed in triplicate using the Brilliant SYBR Green QPCR Core Reagent kit (Stratagene). To ensure amplification of a single homogeneous product, post-amplification dissociation curves were performed. All primer sets produced a single product of expected size (See additional file 3: Figure 1 PCR fragments of genes analysed by QPCR). Changes in gene expression were determined using the standard curve method with β-Actin as the normalizing gene.

**Table 2:** Tissue Cu concentrations and SOD1 activity ^1,2^

Diet Group	Liver Cu (μg/g dry weight)	Kidney Cu (μg/g dry weight)	Mucosal Cu (μg/g dry weight)	Liver SOD1 Activity U/mg protein	Erythrocyte SOD1 Activity U/mg Hb
Zn-30	17.50 ± 0.87^a^	23.08 ± 1.07^a^	9.66 ± 0.25^ab^	27.39 ± 1.90^a^	53.21 ± 2.21^a^
Zn-60	16.08 ± 0.57^ab^	21.28 ± 1.04^ab^	9.21 ± 0.26^ab^	26.91 ± 1.15^a^	52.61 ± 1.97^a^
Zn-120	14.70 ± 1.07^b^	20.57 ± 0.85^b^	8.64 ± 0.51^a^	24.31 ± 1.84^a^	48.58 ± 1.45^a^
Zn-240	16.38 ± 0.65^ab^	21.74 ± 0.82^ab^	9.78 ± 0.36^b^	25.25 ± 1.44^a^	49.06 ± 1.61^a^
Cu-D	9.45 ± 0.82^c^	15.84 ± 0.32^c^	5.50 ± 0.49^c^	19.40 ± 1.76^b^	41.81 ± 1.55^b^

^1 ^Values in a column without a common letter differ, *P *< 0.05.

^2 ^Values are means ± SEM, n = 12/diet group.

**Figure 1:**
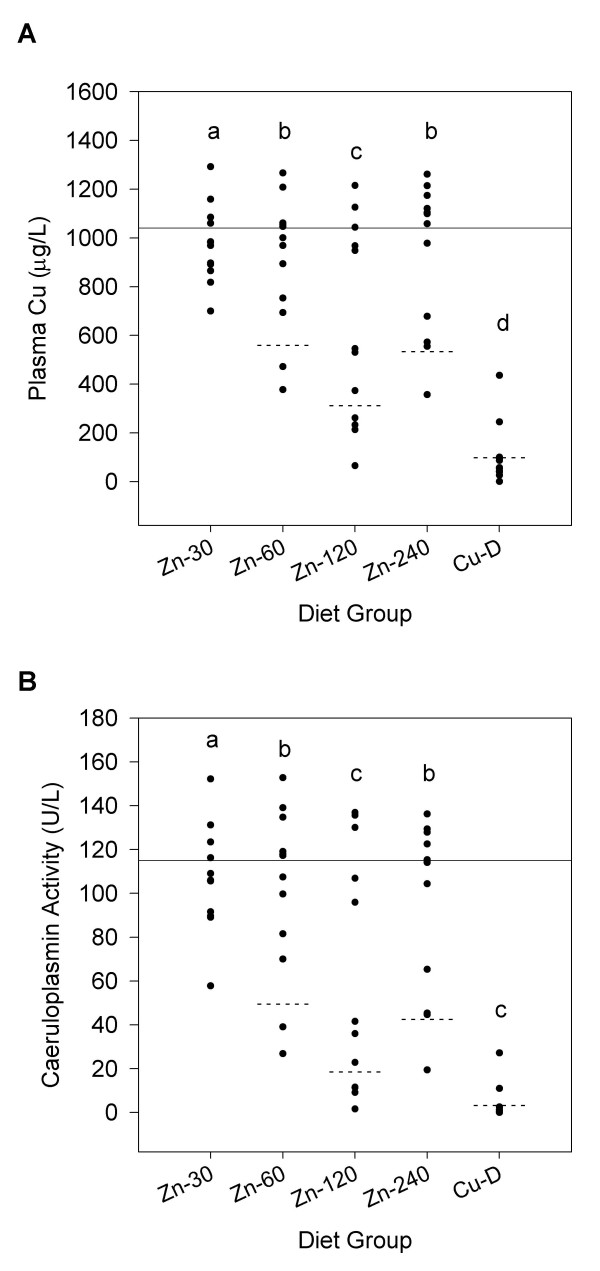
**Scatter plots of (A) plasma Cu concentration and (B) Cp activity of rats fed diets differing in Zn and Cu**. Each solid circle corresponds to one rat (n = 12/diet group). The horizontal line across all diet groups represents the non-response mean of Zn-30 rats and that of the non-responders from other diet groups (see Methods). The dashed line within each diet group signifies the response mean for that diet group. The non-response mean (diet group Zn-30) and the response means (diet groups Zn-60, Zn-120, Zn-240 and Cu-D) were compared. Diet groups without a common letter differ (*P *< 0.05).

### Enzyme Assays

Liver and erythrocyte SOD1 activity was measured by the cytochrome c reduction assay [31] modified for analysis with a microplate reader. Plasma caeruloplasmin (Cp) activity was measured as described [32].

### Statistical Analyses

Data were analysed by one-way ANOVA and differences between means were determined by Fisher's least significant difference test. Data are reported as mean ± SEM. Linear regression analyses were performed to examine the association between conventional indices of Cu status and tissue CCS expression. Pearson's correlation coefficient (r) was calculated to measure the goodness of fit of the data. To test for differences in the response means and the non-response mean for plasma Cu and Cp activity (Figure 1), data were analysed using an ANOVA model with diet group as the main effect. The mean levels were compared using Tukey's Studentized Range Test. Because of the partial response behaviour, these data were fitted using maximum likelihood methods with the diet groups fitted with the same mean as the Zn-30 group for the non-responders and separate means for the responders, each with a common variance and proportion of responders. The response means and the non-response mean were compared using the log-likelihood difference and grouped when not significantly different. Because the maximum likelihood approach does not classify individual observations, a cut-off value of 2 standard deviations from the mean of Zn-30 rats was used to characterise individual rats as responders and non-responders (Table 3). Fisher exact test was used to determine differences in the number of responders between diet groups. Statistical significance was set at *P *< 0.05. Data were analysed using Statistica 7 software (StatSoft, Tulsa, OK).

**Table 3:** Effect of dietary Zn and Cu on plasma Cu concentration and Cp activity of rats

Animals	Plasma Cu Concentration^1,2^					Cp Activity^3,4^				
		
	Zn-30	Zn-60	Zn-120	Zn-240	Cu-D	Zn-30	Zn-60	Zn-120	Zn-240	Cu-D
No. of non-responders	12	10	5	9	0	11	10	5	9	0
No. of responders^5^	0^a^	2^ab^	7^b^	3^ab^	12^c^	1^a^	2^ab^	7^b^	3^ab^	12^c^
Total	12	12	12	12	12	12	12	12	12	12

^1 ^Non-responders had a range of 667.6 – 1291.6 μg/L.

^2 ^Responders (range <0.1 – 572.4 μg/L) were those animals having plasma Cu concentration lower than 2SD below the mean plasma copper concentration of animals in the Zn-30 diet group (Zn-30; mean ± SD = 975.0 ± 159.4 μg/L).

^3 ^Non-responders had a range of 65.3 – 152.8 U/L.

^4 ^Responders (range <0.1 – 57.8 U/L) were those animals having Cp activity lower than 2SD below the mean Cp activity of animals in the Zn-30 diet group (Zn-30; mean ± SD = 106.5 ± 23.9).

^5 ^No. of responders were compared using the Fisher exact test. For each diet group, No. of responders without a common letter differ, *P *< 0.05.

## Results

Test diets were prepared by adding 30, 60, 120 and 240 mg Zn/kg diet, which corresponds to normal amounts of Zn or 2, 4 or 8 times the AIN recommended amount [33], respectively. An additional group of rats was fed a Cu-deficient diet containing normal amounts of Zn and served as a positive control for Cu deficiency. Precise Zn and Cu content of each test diet is shown in Table 1. All test diets contained higher amounts of Zn than what would be expected from amounts added to the diet preparations. Analysis of individual dietary components revealed that casein was the major contributor to the additional Zn in the final diet preparations (data not shown).

Total food consumption of rats from each diet group was similar (Table 1). Rats fed the Zn-120 and Zn-240 diet accumulated larger (*P *< 0.05) amounts of Zn in the liver compared to control rats fed normal amounts of Zn (Zn-30) (Table 1). Only rats fed the largest amount of Zn (Zn-240) showed a significant (*P *< 0.05) increase in kidney Zn content. Zn in the intestinal mucosa of rats fed the Zn-120 or the Zn-240 diet was increased (*P *< 0.05). Zn content in the liver, kidney and intestinal mucosa of rats fed the Cu-deficient diet (Cu-D) was similar to that of rats fed adequate Cu (Zn-30), indicating that the Cu-deficient diet did not affect Zn accumulation in these tissues.

Rats consuming the diet containing the largest amount of Zn (Zn-240) showed a significant (*P *< 0.05) increase in body weight at week 4 (data not shown) and 5 (Table 1) of the study compared to rats fed normal Zn. Conversely, body weight of rats fed the Zn-60, Zn-120 or Cu-D diet did not differ from that of Zn-30 rats (Table 1). Haemoglobin (Hb) levels and other haematological parameters of rats fed elevated Zn or the Cu-D diet were similar to those of rats fed the Zn-30 diet (See additional file 1: Table 1).

Rats fed 4 times the normal level of Zn (Zn-120) showed a small decrease (*P *< 0.05) in liver Cu compared to rats fed normal Zn (Table 2). Surprisingly, liver Cu did not decrease further in rats fed the highest amount of Zn (Zn-240). In fact, Cu levels did not differ from those of Zn-30 rats. A similar trend was observed for kidney Cu content. Diets high in Zn did not alter Cu content in the intestinal mucosa when compared to rats fed normal Zn. However, Cu content in the intestinal mucosa was higher (*P *< 0.05) in Zn-240 compared to Zn-120 rats. Cu levels in the liver, kidney and intestinal mucosa of rats fed the Cu-D diet were markedly decreased (*P *< 0.05) compared to rats fed the control diet. Liver and erythrocyte SOD1 activity was unaffected (*P *> 0.05) in rats fed high Zn (Table 2). In contrast, rats fed the Cu-D diet showed a significant (*P *< 0.05) decrease in SOD1 activity in liver and erythrocytes.

Scatter plots of plasma Cu levels clearly showed that while some rats responded to increased dietary Zn with a reduction in plasma Cu other rats had normal levels (Figure 1A), indicating an all or none response. The number of rats that responded increased from 2 rats when fed the Zn-60 diet to 7 rats when fed the Zn-120 diet (Table 3). Consistent with liver, kidney and intestinal mucosa Cu content indicating improved Cu status in rats fed the Zn-240 diet compared to rats fed the Zn-120 diet, only 3 rats fed the Zn-240 diet were characterised as responders. The response mean for plasma Cu was also higher in Zn-240 compared to Zn-120 rats (Figure 1A). Plasma Cu of Cu-D rats was markedly decreased, consistent with data from a previous study from our laboratory [10]. As expected, given that most plasma Cu is associated with Cp, Cp activity paralleled very closely plasma Cu levels and showed a similar all or none response behaviour (Figure 1B, Table 3). A strong positive correlation (r = 0.984) was found between plasma Cu and Cp activity (data not shown).

**Figure 2:**
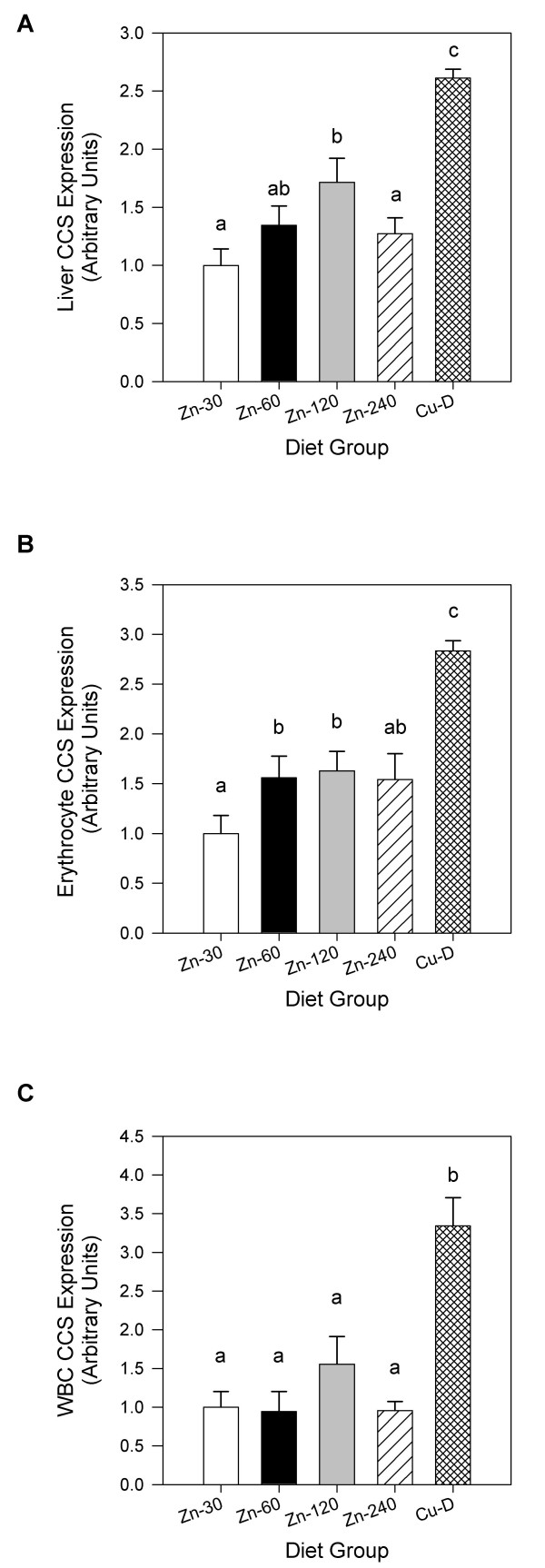
**(A) Liver, (B) erythrocyte and (C) WBC CCS content of rats fed diets differing in Zn and Cu**. CCS expression in liver is expressed relative to that of β-Actin. CCS expression in erythrocytes and WBCs is expressed relative to that of GAPDH. Bars signify the mean ± SEM, n = 12 (for liver and erythrocytes) or 7 (for WBCs)/diet group. Diet groups without a common letter differ (*P *< 0.05).

Liver CCS was increased (*P *< 0.05) >1.7-fold in rats fed the Zn-120 diet compared to rats fed normal Zn (Figure 2A). CCS expression was lower (*P *< 0.05) in Zn-240 rats compared to Zn-120 rats. Erythrocyte CCS was increased (*P *< 0.05) >1.5-fold in Zn-60 and Zn-120 rats (Figure 2B). The Cu-D diet induced a larger increase in CCS content in liver and erythrocytes (>2.5-fold). WBC CCS expression was not significantly (*P *> 0.05) increased in rats fed elevated Zn, but was increased (*P *< 0.05) >3-fold in Cu-D rats (Figure 2C). Rats fed high Zn and characterised as responders for plasma Cu had higher (*P *< 0.05) liver and WBC CCS expression than non-responders or Zn-30 rats (Table 4). Erythrocyte CCS expression of responders was higher (*P *< 0.05) than that of Zn-30 rats, but not significantly (*P *> 0.05) different from non-responders.

**Table 4:** Comparison of tissue CCS expression between Zn-30 rats and responders and non-responders for plasma Cu^1,2^

Animals	CCS Expression		
	
	Liver	Erythrocyte	WBC
Zn-30	1.00 ± 0.14^a ^ (n = 12)	1.00 ± 0.18^a ^ (n = 12)	1.00 ± 0.20^a ^ (n = 7)
Non-responders^3^	1.18 ± 0.11^a ^ (n = 24)	1.44 ± 0.16^ab ^ (n = 24)	0.95 ± 0.11^a ^ (n = 17)
Responders^4^	1.97 ± 0.12^b ^ (n = 12)	1.86 ± 0.20^b ^ (n = 12)	2.02 ± 0.54^b ^ (n = 4)

^1 ^Values in a column without a common letter differ, *P *< 0.05. Values are means ± SEM.

^2 ^n values are in parentheses.

^3 ^Rats from diet groups Zn-60, Zn-120 and Zn-240 characterised as non-responders for plasma Cu.

^4 ^Rats from diet groups Zn-60, Zn-120 and Zn-240 characterised as responders for plasma Cu.

Pearson linear correlations revealed a strong inverse association between liver CCS and liver (r = -0.746) and plasma (r = -0.816) Cu and Cp activity (r = -0.787) (Table 5). A significant (*P *< 0.001) association was also observed when rats from the Cu-D diet group were omitted from the analyses. Liver CCS only showed a moderate inverse correlation with liver and erythrocyte SOD1 activity when all rats were used in the analyses. A significant correlation was not found (*P *> 0.05) when Cu-D rats were omitted from the analyses, consistent with the lack of a significant decrease in SOD1 activity in rats fed elevated Zn (Table 2). Erythrocyte CCS expression was also significantly (*P *< 0.001) correlated with liver and plasma Cu and Cp activity (Table 5). WBC CCS expression showed a strong inverse correlation with liver (r = -0.768) and plasma (r = -0.813) Cu and Cp activity (r = -0.769).

**Table 5:** Correlation between CCS content in tissues and liver and plasma Cu concentration, Cp activity and liver and erythrocyte SOD1 activity^1,2^

Tissue CCS	Diet Groups^3^, n = 60					Diet Groups^4^, n = 48				
		
	Liver Cu	Plasma Cu	Cp	Liver SOD1	Erythrocyte SOD1	Liver Cu	Plasma Cu	Cp	Liver SOD1	Erythrocyte SOD1
Liver	-0.746 (<0.001)	-0.816 (<0.001)	-0.787 (<0.001)	-0.418 (<0.005)	-0.464 (<0.001)	-0.597 (<0.001)	-0.649 (<0.001)	-0.615 (<0.001)	-0.222 (NSD)	-0.221 (NSD)
Erythrocyte	-0.633 (<0.001)	-0.618 (<0.001)	-0.590 (<0.001)	-0.268 (<0.05)	-0.332 (<0.01)	-0.425 (<0.005)	-0.308 (<0.05)	-0.297 (<0.05)	-0.001 (NSD)	0.001 (NSD)
WBC	-0.768^5 ^ (<0.001)	-0.813^5 ^ (<0.001)	-0.769^5 ^ (<0.001)	-0.495^5 ^ (<0.005)	-0.582^5 ^ (<0.001)	-0.474^6 ^ (<0.05)	-0.559^6 ^ (<0.005)	-0.543^6 ^ (<0.005)	-0.069^6 ^ (NSD)	-0.429^6 ^ (<0.05)

^1 ^Pearson's correlation coefficients.

^2 ^*P *values are in parentheses; NSD = no statistical difference.

^3 ^Diet groups Zn-30, Zn-60, Zn-120, Zn-240, and Cu-D.

^4 ^Diet groups Zn-30, Zn-60, Zn-120, and Zn-240.

^5 ^n = 35.

^6 ^n = 28.

**Figure 3:**
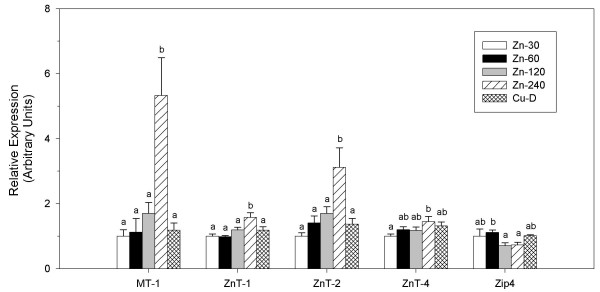
**QPCR analysis of duodenal mRNA expression of Zn-trafficking proteins in rats fed diets differing in Zn and Cu**. MT-1, ZnT-1, ZnT-2, ZnT-4 and Zip4 mRNA content is expressed relative to β-Actin expression. Bars represent the mean ± SEM, n = 4/diet group. For each gene, diet groups without a common letter differ (*P *< 0.05).

Given that rats fed the highest amount of Zn (Zn-240) showed better Cu status than rats fed a lower level of excess Zn (Zn-120), it prompted us to evaluate changes in expression of duodenal Zn-trafficking proteins in response to these levels of Zn. Messenger RNA for MT-1 and Zn transporters ZnT-1, ZnT-2 and ZnT-4 was significantly (*P *< 0.05) increased only in Zn-240 rats when compared to Zn-30 rats (Figure 3). MT-1 (~5-fold) and ZnT-2 (~3-fold) showed the largest increases followed by ZnT-1 and ZnT-4. Zip4 expression was unchanged in rats fed elevated Zn compared to rats fed normal Zn. Collectively, these data indicate that mRNA expression of MT-1 and several ZnT transporters in the duodenum of rats is refractory to small increases in dietary Zn, but responds to a higher level of Zn intake.

## Discussion

This is the first study to demonstrate that CCS protein expression responds to mild Cu deficiency induced by moderately high dietary Zn, underscoring the potential usefulness of CCS as a biomarker in a clinical setting. In contrast to rats and mice used in previous studies [19,21,22], rats used in this study had normal haematology and did not show any indications of the development of anaemia, indicating that these rats were not overtly Cu deficient. Further, rats did not show impaired growth, a phenotype associated with severe Cu deficiency in growing rats [19].

Erythrocyte CCS expression was determined to be the most sensitive index of reduced Cu nutriture and was able to detect a decrease in Cu status in rats fed only twice the AIN recommended amount of Zn [33]. This sensitivity may also partly account for the absence of a significant difference in erythrocyte CCS expression between responders and non-responders for plasma Cu, as CCS may have increased in some rats fed high Zn but characterise as non-responders. Notably, erythrocyte CCS expression in non-responders was higher than in Zn-30 rats and this difference was approaching statistical significance (*P *= 0.09). Liver CCS also responded to elevated dietary Zn and was increased in rats fed 4 times the recommended amount. These are important findings, as these same rats did not show a significant decrease in SOD1 activity, the biomarker of reduced Cu status used to set the ULs for Zn.

Rats fed elevated Zn showed an all or none response for decreased plasma Cu and Cp activity, indicating that these parameters are not ideal for diagnosing small reductions in Cu status and providing an accurate assessment of precise Cu nutriture. These data are consistent with a previous study showing a similar all or none response for Cp activity in rats fed elevated levels of Zn [9]. This response likely reflects the presence of strong homeostatic mechanisms that maintain plasma Cu levels in the normal range until liver stores have been appreciably depleted.

Although a gold standard for assessing reductions in Cu status is lacking, decreased liver Cu content is regarded as one of the most accurate and sensitive measures in experimental animals, while plasma Cu and Cp activity are the most common indicators used in clinical situations. The strong inverse correlation of liver CCS expression with these markers indicates that increased CCS expression was specific for reduced Cu status and is a good predictor of changes in common measures of Cu deficiency. Erythrocyte CCS showed a weaker association with these indicators that may be explained, in part, by the slow turnover of erythrocytes, which may not accurately reflect the current Cu status of the rats. This is in contrast to plasma and liver Cu levels and Cp activity that likely respond more rapidly to changes in Cu availability.

During our experiments we discovered that CCS protein in WBCs is more susceptible to degradation than CCS in erythrocytes, requiring that WBCs be isolated quickly from blood samples. Samples showing degradation of CCS were excluded from our analyses. Of the 7 rats per diet group analysed, we noticed that the majority of rats fed high Zn were characterised as non-responders for decreased plasma Cu and Cp activity. This over sampling of rats showing normal plasma Cu and Cp activity may account for the absence of a significant increase in WBC CCS in rats fed elevated Zn. Nonetheless, WBC CCS expression was increased in rats fed the Cu-deficient diet and was highly correlated with conventional measures of Cu status. Further, rats characterised as responders for plasma Cu (which represents rats with poorer Cu status) had higher WBC CCS levels than non-responders or Zn-30 rats, indicating that WBC CCS responds to Cu deficiency induced by excess zinc. Because of the slow turnover of erythrocytes, WBC CCS may have value as an indicator of early reductions in Cu status. Interestingly, we found that the basal expression level of CCS in WBCs is much higher than in other tissues such as liver, heart and erythrocytes in rats (data not shown).

A major finding of this paper is that rats fed 8 times the recommended amount of Zn (Zn-240) showed overall better Cu status than rats fed 4 times the recommended amount (Zn-120). This was consistently observed with several biomarkers. Remarkably, Cu status indicators of most rats fed the Zn-240 diet were indistinguishable from rats fed a diet normal in Zn. Importantly, liver CCS was significantly lower in Zn-240 compared to Zn-120 rats, further demonstrating the exquisite sensitivity of CCS to changes in Cu status. At this point, we cannot offer a definite explanation as to why other studies from our laboratory [9,34] failed to detect this biphasic response, other than to speculate that differences in the bioavailability of the Zn source, other dietary components or duration of the studies may account for this discrepancy.

Presently, the mechanism by which Zn interferes with Cu absorption is unknown, although it is believed to occur at the site of the intestinal enterocyte. It was thought that increased metallothionein (MT) levels sequestered Cu in the enterocyte leading to Cu deficiency [35,36]. However, MT-null mice fed elevated Zn become Cu deficient indicating that MT induction is not the primary cause of the Cu deficiency [37]. Results presented here are consistent with these data, as rats fed the Zn-60 and Zn-120 diet showed reduced Cu status in the absence of increased duodenal MT-1 transcript. Because MT can bind Cu, however, it would be interesting to determine whether higher MT-1 expression in Zn-240 rats played any role in increasing Cu in the intestinal mucosa and improving Cu status of these rats compared to Zn-120 rats.

Experiments with Caco-2 cells have indicated that elevated levels of Zn affect Cu transport and reduce Cu efflux at the basolateral side [38], suggesting that Zn may block absorption of Cu by affecting the activity of a Cu transporter. Thus, improved Cu status of Zn-240 compared to Zn-120 rats may reflect a secondary effect of the higher Zn diet on the activity of a Cu transporter. It is known that distinct pools of Zn exist within cells and changes in expression of Zn-trafficking proteins can alter the intracellular distribution of Zn [39]. For example, increased expression of ZnT-2 likely promotes the accumulation of Zn within vesicles, decreasing cytoplasmic Zn levels [40]. In addition, increased MT levels result in more Zn bound to MT. Given that transcripts of several Zn-trafficking proteins were increased in the duodenum of rats fed the Zn-240 but not the Zn-120 diet, it is possible that increased expression of one or more of these Zn-trafficking proteins altered the distribution of Zn within absorptive enterocytes of Zn-240 rats in a manner that alleviated the block in activity of a Cu transporter.

Lastly, we report an interesting observation that rats fed the highest level of Zn had increased body weight. Although it is well recognized that Zn deficiency can result in impaired growth [41], it is not established that consuming larger quantities of Zn, above recommend amounts, can enhance growth. Given that lower doses of Zn above the AIN recommended amount clearly depressed Cu status of rats, the benefit of consuming this higher level of Zn is presently unclear.

## Conclusion

In this study we report two major discoveries. Firstly, we show that CCS is a sensitive biomarker of Zn-induced mild Cu deficiency in rats. Given this finding, further studies evaluating CCS as a biomarker in humans are necessary, as CCS may prove to be a better measure of reduced Cu status than conventional indicators and impact studies aimed at setting more accurate Dietary Reference Intakes for Zn and Cu. Secondly, we demonstrate a dose-dependent biphasic response for the reduction of Cu status by moderately high intakes of Zn. Although it is not known whether humans respond to excess Zn in a similar manner to rats, this finding offers a caution when interpreting data from studies reporting no reduction in Cu status that have used a single dose of supplemental Zn and emphasizes the importance of using a dose-response approach.

## Competing interests

The author(s) declare that they have no competing interests.

## Authors' contributions

MI performed the QPCR and statistical analyses. MI and ES carried out the tissue mineral analyses and the determination of tissue CCS expression. ES performed the enzyme assays. KT prepared the test diets and participated in the animal phase of the study. BW isolated the blood components. ML participated in planning the study and was involved in the interpretation of the results. JB wrote the manuscript and was involved in planning and coordinating the study and interpreting the data. All authors read and approved the final manuscript.

## Supplementary Material

Additional file 1 White blood cells (WBC), erythrocytes (ERCS), haemoglobin (Hb), haematocrit (HCT), mean corpuscular volume (MCV), mean corpuscular haemoglobin (MCH), mean corpuscular haemoglobin concentration (MCHC), reticulocytes (RTC), platelet count (PLT) and red cell distribution width (RDW) of weanling male Wistar rats fed diets with differing amounts of Zn and Cu for 5 weeks (Table 1). Click here for file

Additional file 2 QPCR primers (Table 2). Click here for file

Additional file 3 PCR fragments of genes analysed by QPCR (Figure 1). Click here for file
