# Supplementary material for: Copper Chaperone for Cu/Zn Superoxide Dismutase is a sensitive biomarker of mild copper deficiency induced by moderately high intakes of zinc
Source: Nutr J. 2005 Nov 24;4:35. doi: 10.1186/1475-2891-4-35 (PMC1315358; doi:10.1186/1475-2891-4-35)
Supplement: Additional file 1 — White blood cells (WBC), erythrocytes (ERCS), haemoglobin (Hb), haematocrit (HCT), mean corpuscular volume (MCV), mean corpuscular haemoglobin (MCH), mean corpuscular haemoglobin concentration (MCHC), reticulocytes (RTC), platelet count (PLT) and red cell distribution width (RDW) of weanling male Wistar rats fed diets with differing amounts of Zn and Cu for 5 weeks (Table 1). [file 1475-2891-4-35-S1.pdf]

Table 1: White blood cells (WBC), erythrocytes (ERCS), haemoglobin (Hb), haematocrit (HCT), mean corpuscular volume (MCV), mean corpuscular haemoglobin (MCH), mean corpuscular haemoglobin concentration (MCHC), reticulocytes (RTC), platelet count (PLT) and red cell distribution width (RDW) of weanling male Wistar rats fed diets with differing amounts of Zn and Cu for 5 weeks[1]

Diet Group[2]	WBC	ERCS	Hb	HCT	MCV	MCH	MCHC	RTC	PLT	RDW
	10^9/L	10^{12}/L	g/L	%	fl	pg	g/L	%	10^9/L	%
Zn-30	5.2 ± 0.5^a	6.8 ± 0.1^a	132.8 ± 1.6^a	40.08 ± 0.51^a	58.5 ± 0.6^a	19.6 ± 0.2^a	332.8 ± 1.9^a	3.66 ± 0.21^a	1102 ± 33^a	12.5 ± 0.3^{ab}
Zn-60	5.5 ± 0.6^a	6.8 ± 0.1^a	133.6 ± 1.7^a	40.17 ± 0.61^a	59.1 ± 0.5^a	19.8 ± 0.1^a	332.4 ± 1.7^a	3.64 ± 0.09^a	1118 ± 29^a	12.2 ± 0.2^a
Zn-120	5.6 ± 0.5^a	6.7 ± 0.1^a	130.8 ± 1.1^a	39.33 ± 0.45^a	58.5 ± 0.6^a	19.6 ± 0.2^a	332.8 ± 2.5^a	3.71 ± 0.13^a	1176 ± 46^a	12.4 ± 0.2^{ab}
Zn-240	5.0 ± 0.6^a	6.8 ± 0.1^a	132.3 ± 1.2^a	39.92 ± 0.43^a	58.7 ± 0.6^a	19.4 ± 0.2^a	331.6 ± 1.7^a	3.71 ± 0.16^a	1094 ± 26^a	12.2 ± 0.1^a
Cu-D	5.0 ± 0.4^a	6.9 ± 0.1^a	130.2 ± 1.2^a	39.83 ± 0.55^a	57.6 ± 0.5^a	18.8 ± 0.2^a	327.0 ± 2.2^a	3.65 ± 0.21^a	1127 ± 40^a	12.9 ± 0.2^b

[1] Values are means ± SEM, n = 12/diet group. Values in a column without a common letter differ, *P* < 0.05.
