# Supplementary material for: Copper Chaperone for Cu/Zn Superoxide Dismutase is a sensitive biomarker of mild copper deficiency induced by moderately high intakes of zinc
Source: Nutr J. 2005 Nov 24;4:35. doi: 10.1186/1475-2891-4-35 (PMC1315358; doi:10.1186/1475-2891-4-35)
Supplement: Additional file 2 — QPCR primers (Table 2). [file 1475-2891-4-35-S2.pdf]

Table 2: QPCR primers

Gene (GenBank accession no.)		Primer Sets (5′ – 3′)	Product Size (bp)
ZnT-1	Forward	ACC AGG CAG AGC CAG AGA AGT T	226
(U17133)	Reverse	TCG TCT TCA GTA CAA CCC TTC CAG G	
ZnT-2	Forward	GCG CTG TGG CTG TGA ACA TCA TAA	137
(NM_012890)	Reverse	AAC CAC ATG AAT GAA GGC AGC TCG	
ZnT-4	Forward	TAG TGC ACA TGC AGC TAA TTC CCG	258
(NM_172066)	Reverse	TCA GGG ACT CCA TTA GGT TTG CGT	
Zip4	Forward	CAA TAT CAC GCT GCC CGA ATT GGA	203
	Reverse	CAG CCA TTA CAT CTT TGG CAC TCA GG	
MT-1	Forward	TAC ACC GTT GCT CCA GAT TCA CCA	255
(NM_138826)	Reverse	TAT TTA CAC CTG AGG GCA GCA GCA	
β-Actin	Forward	AGG TCA TCA CTA TCG GCA ATG AGC	164
(NM_031144)	Reverse	AGA CAG CAC TGT GTT GGC ATA GAG	
