# Supplementary material for: Copper Chaperone for Cu/Zn Superoxide Dismutase is a sensitive biomarker of mild copper deficiency induced by moderately high intakes of zinc
Source: Nutr J. 2005 Nov 24;4:35. doi: 10.1186/1475-2891-4-35 (PMC1315358; doi:10.1186/1475-2891-4-35)
Supplement: Additional file 3 — PCR fragments of genes analysed by QPCR (Figure 1). [file 1475-2891-4-35-S3.pdf]

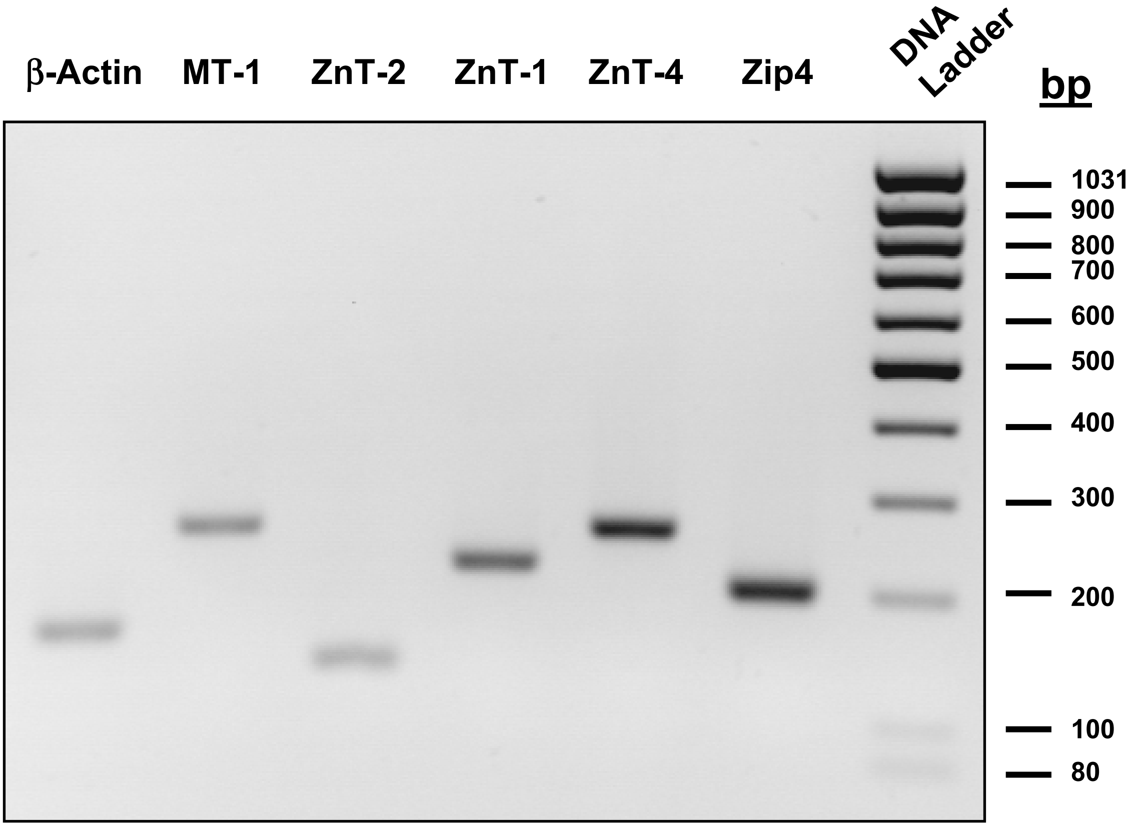

Figure 1: PCR fragments of genes analysed by QPCR. PCR fragments of the rat *β-Actin*, *MT-1*, *ZnT-1*, *ZnT-2*, *ZnT-4* and *Zip4* genes were amplified from total RNA isolated from rat duodenum using the QIAGEN OneStep RT-PCR Kit with the primer sets used for QPCR analyses. PCR products were run on a 1.5% agarose gel and visualized with ethidium bromide staining. Sizes of DNA fragments of the Low Range MassRuler^{TM} DNA Ladder (Fermentas, Burlington, Canada) are shown to the right.
